# DisSAGD: A Distributed Parameter Update Scheme Based on Variance Reduction

**DOI:** 10.3390/s21155124

**Published:** 2021-07-28

**Authors:** Haijie Pan, Lirong Zheng

**Affiliations:** School of Information Science and Engineering, Fudan University, Yangpu District, Shanghai 200433, China; 17110720052@fudan.edu.cn

**Keywords:** gradient descent, machine learning, distributed cluster, adaptive sampling, variance reduction

## Abstract

Machine learning models often converge slowly and are unstable due to the significant variance of random data when using a sample estimate gradient in SGD. To increase the speed of convergence and improve stability, a distributed SGD algorithm based on variance reduction, named DisSAGD, is proposed in this study. DisSAGD corrects the gradient estimate for each iteration by using the gradient variance of historical iterations without full gradient computation or additional storage, i.e., it reduces the mean variance of historical gradients in order to reduce the error in updating parameters. We implemented DisSAGD in distributed clusters in order to train a machine learning model by sharing parameters among nodes using an asynchronous communication protocol. We also propose an adaptive learning rate strategy, as well as a sampling strategy, to address the update lag of the overall parameter distribution, which helps to improve the convergence speed when the parameters deviate from the optimal value—when one working node is faster than another, this node will have more time to compute the local gradient and sample more samples for the next iteration. Our experiments demonstrate that DisSAGD significantly reduces waiting times during loop iterations and improves convergence speed when compared to traditional methods, and that our method can achieve speed increases for distributed clusters.

## 1. Introduction

Big data are often the motivating force driving the development of artificial intelligence, especially in image recognition, semantic recognition, and text information processing. These developments often achieve excellent results [1]; however, deep learning models have many parameters and rely on learning rules from big data [2]. For example, by providing terabytes of training data to large-scale deep neural networks for model training, their trained deep neural networks end up with millions or billions of parameters. Since many machine learning algorithms converge through iterations, they require multiple rounds of iterative computation to update the model’s parameters. Finding an efficient training method is crucial to performing large-scale machine learning tasks due to the complexity of machine learning models and the amount of training data and computations required [3].

The objective function of deep learning can generally be described as an optimization problem [4] as shown in Equation (1):

(1) $$\min f\left(\omega \right),\;f\left(\omega \right)=-\sum f_{i}\left(\omega \right)+R\left(\omega \right)$$

where f(ω) is loss, $f_{i}\left(\omega \right)$ in which 1≤i≤m denotes the *i*th data point. ω denotes the model parameters, i.e., the parameters to be updated during the iteration, and *m* denotes the size of the training data. R(ω) denotes the regularization term used to avoid overfitting, which usually is the L1 parametric when a sparse solution needs to be obtained, e.g., when the optimal parameters contain many zeros, and the ridge regression model uses the L2 parametric as the regularization term. In this case, the machine learning model training process minimizes the loss function by iteratively updating the parameters.

Traditionally, the model learning process uses gradient descent for model parameter updates, i.e., $\omega_{t}=\omega_{t-1}-\lambda_{t-1}\nabla f\left(\omega_{t-1}\right)$, where *t* denotes the *t*th iteration, and λ is the learning rate [5]. When *m* training data samples are sampled in each iteration, *m* computational derivations are required for $\nabla f\left(\omega_{t-1}\right)$, making the gradient descent method impractical. The derivation is time consuming when *m* is large. Time consuming derivation problem can be solved by using SGD, where a random sample of the training data can be used to compute the local gradient $\nabla f_{i}\left(\omega_{t-1}\right)$. Then the local gradient is used to update model parameters. Since $\nabla f_{i}\left(\omega_{t-1}\right)$, only one derivation of the local gradient is required during the iteration, which is effective for large-scale machine learning tasks; however, the variance between the local gradient $\nabla f_{i}\left(\omega_{t-1}\right)$ and the global average gradient $\nabla f\left(\omega_{t-1}\right)$ can slow down model training convergence. In this paper, random noise is used to equivalently represent the variance, which in turn $\nabla f_{i}\left(\omega_{t-1}\right)$ can be expressed as a “noisy” gradient estimate for the t−1th iteration chosen at random. This rough approximation of the gradient is convenient and efficient when there is a large variance and can be quickly converged in solutions with relatively low accuracy requirements; however, even when the gradient solution approaches the saddle point, the large variance hinders continued loss function reduction.

To address this problem, some scholars have reduced the variance by updating the parameters by using a decaying learning rate [6,7,8], i.e., the learning rate decreases continuously as the iterations increase. Although this approach can reduce the variance, it can affect other aspects, e.g., the decreased learning rate makes it difficult for the model to converge. Many scholars have also proposed accelerating the convergence of SGDs by variance reduction (VR) schemes [9,10], which enable the fast finding of saddle points in the gradient descent solution and result in a fast and stable model convergence; however, improved SGDs are intended to be used on a centralized single node rather than a cluster of working nodes. It is important to note that variance reduction techniques do not help when there are so many model parameters that the model cannot be trained on the hardware nodes. For example, deep networks may have billions or even trillions of parameters, resulting in improved SGDs that cannot effectively be trained for tasks with large amounts of data or model parameters, which is a challenge that urgently needs to be addressed.

Moreover, since SGD requires constant iterations over the training data and traverses the dataset many times, the algorithm execution is often inefficient for large datasets and when using a single machine with limited computational power [11]. SGDs and their variants are used in a distributed form to perform coordinated updates of model parameters [12,13] to address these problems in the context of massive data. Distributed SGDs usually train machine learning models and update parameters in distributed clusters. Generally, the nodes of a distributed cluster are structured in servers and working nodes. First, the working nodes extract the server’s parameters, and secondly, the basic computation of the gradient is performed by the working nodes. These updates are then pushed to the server and aggregated and averaged to update the global parameters resulting in updated global parameters that will be made available to the working nodes. Nowadays, distributed stochastic gradient descent algorithms are widely studied, and improving their convergence speed and performance has important applications [14,15,16].

This paper proposes a distributed gradient descent optimization algorithm DisSAGD (distributed stochastic average gradient descent). DisSAGD is based on the average gradient vector variance reduction for massive data, which does not require full gradient computation or additional storage. In this paper, DisSAGD was set to be asynchronous and accelerated, using an adaptive learning rate to achieve fast convergence. There is a “lag problem” in server global parameter distribution when performing iterative computation in distributed clusters. Such fast working nodes will spend a lot of time waiting for slow working nodes to obtain global parameters, which means that the fast working nodes idle when waiting for slow working nodes to finish computation. The number of samples can be set based on the model’s training level in order to reduce waiting time. To achieve this, we propose an adaptive sample size variation method to solve this challenge. We set the adaptive sample size variation method to dynamically adjust the number of training samples during the model training iterations. For example, if a node computes faster than other nodes, it is set to increase the number of samples for the next iteration of training so that the faster node can compute more local gradients. This allows the slower nodes to catch up with the faster nodes to achieve synchronization, resulting in a significant reduction in average waiting times of nodes. Our experimental results show that DisSAGD outperforms other current versions of distributed SGD.

The contributions of this paper are as follows:

* To solve the diminishing dynamic function issue that causes the model to converge quickly when there is a large variance in the distributed scenario SGD. To this end, we propose a distributed gradient descent optimization algorithm—DisSAGD—which is based on a mean gradient vector variance reduction for large-scale machine learning tasks that uses a mean gradient vector variance reduction-based technique to update the parameters in the model. It does not require full gradient computation or additional storage, as the DisSAGD uses an asynchronous approach to achieve a reasonable DisSAGD convergence speed, compared to other versions of distributed SGDs.
* To address the node “lag problem” in distributed clusters, we propose an adaptive sampling strategy that dynamically adjusts the random sampling strategy during the iteration, so that when a working node is faster than other working nodes, it will increase the amount of samples for the next iteration, which allows faster working nodes more time to compute the local gradient. As a result, the slow working nodes have a chance to catch up to the fast working nodes, resulting in a significant reduction in average node waiting times.
* Variance reduction techniques can exploit some intrinsic properties to accelerate the convergence of machine learning algorithms; therefore, we propose an adaptive learning rate with an acceleration factor that consists of two elements: the fixed value (constant) and the acceleration factor. The constant is obtained from historical experience, i.e., from the local optimum. The variation of the acceleration factor is adjusted based on the distance of the parameter from the optimal value.
* In the experimental training for anomaly detection and classification regression task scenarios for HPC clusters, our performance evaluation proves that DisSAGD outperforms other versions of distributed SGDs with faster convergence, showing that our method can effectively solve the lag problem and reduce the waiting time between nodes.

The remainder of this article is organized as follows. In Section 2, we discuss the related literature of distributed stochastic gradient descent algorithms based on variance reduction. In Section 3, we present the variance reduction method and discuss the shortcomings of the previous SVRG method. In Section 4, the implementation of DisSAGD in a distributed machine learning cluster is introduced, describing the execution of client and server-side processes, as well as the overall implementation process. In Section 5, we present our optimization scheme for distributed machine learning clusters, i.e., the adaptive learning rate strategy and sampling. In Section 6, we describe our experiments and compare the performance of the convergence effect, acceleration effect, and waiting time with the baseline. Finally, Section 7 concludes this article, and provides possible future research directions.

## 2. Literature Review

Due to the proliferation of data, distributed stochastic gradient descent algorithms have become a research hotspot in recent years for dealing with big data problems, as they can train large-scale machine learning models using large amounts of training data [17]. In this paper, we focus on distributed stochastic gradient descent algorithms based on variance reduction; for simplicity, we do not discuss related works such as stochastic gradient descent algorithms under shared memory multi-core parallelism [18,19,20] and model-based parallelism [21,22].

The authors of [23] investigate the performance bottlenecks of MLlib (an official Spark package for ML) by analyzing its implementation of stochastic gradient descent (SGD), which is responsible for training in many ML models. The authors of [24] detail two approaches that implement scattering projection to achieve the comprehensive detection of scatterers: (1) controlling the training speed of each node through elastic parallel control; and (2) shifting the blocked tasks from scatterers to pioneers to fully utilize the computational resources [25]. The authors of [26] study federated machine learning at the wireless edge, where power and bandwidth-limited wireless devices with local datasets carry out distributed stochastic gradient descent (DSGD) with the help of a parameter server (PS). In [27], PetuumSGD was proposed, which reduces the variance by reducing the learning rate during the training process; however, when the learning rate is reduced, it results in an increase in model training time. Recently, ref. [28] proposed a new type of distributed SGD, called SSGD, which is implemented by the fusing proximal gradient with the random variance reduction gradient. Although SSGD uses a variance reduction technique, it does not adequately address the node waiting times—the DisSAGD method proposed in this paper can solve this problem much more effectively. In addition, there are some studies that investigate distributed machine learning. For example, ref. [29] proposed FlexRR to solve the problem of slow training due to the lag problem of machine learning algorithms in distributed environments. In the face of rapid parameter growth, which leads to high parameter synchronization costs and greatly slows down distributed learning, ref. [30] proposed a sufficient factor broadcast (SFB) computational model for the efficient distributed learning of large-scale matrix parameterized models. Communication efficiency is improved by broadcasting SF between working nodes and updating matrix parameters, which are locally reconstructed at each node. The authors of [31] proposed a small batch training method to cluster different SGDs to accelerate training, and [32] proposed an error compensation method for the random gradient descent method to improve the training efficiency, and then quantified the interactive information to reduce the communication overhead. In [33], the authors implemented a variance reduction for SGD, but they used the serial data method and had no distributed implementation; therefore, it cannot meet the distributed scenario requirements. Some studies have implemented distributed SVRG [28,34].

These distributed SVRG implementations set multiple learning threads to update parameters in parallel on a single node; however, such work is designed for multi-core systems on a single node. When the implementation scenario needs to be on multiple nodes, these distributed SVRGs cannot be applied; therefore, the variance reduction SGD proposed in this paper meets the requirement of real distributed scene, i.e., there are multiple distributed nodes, which can divide the data into different working nodes so that the collaborative optimization can accelerate SGD. Recently, other improved variance reduction SGDs such as SAG [35], SAGA [36], S2GD, SVRG ++, and Prox-SVRG [37]. All these work havs effectively improved the variance reduction techniques and can be utilized to improve the methods in this paper; however, we will discuss these methods in future work.

To demonstrate the performance advantages of our work with related literature, we summarize the results in Table 1, comparing the speed of convergence, stability, small estimation error, model accuracy and amount of communication data; our work can satisfy each performance metric.

## 3. Variance Reduction

Several variance reduction methods use multiple iterations during machine model training, with each iteration traversing the entire dataset [28]; thus, *n* updates (one update per data record/feature vector) occur during the *t*th iteration, whose generated iterative model parameters can be expressed as ${\left\{\omega_{t}^{j}\right\}}_{j=1}^{n}$. For comparison, at the end of each iteration, SVRG is set to $y=\omega_{t}^{n}$, and by using ${\bar{g}}_{y}=\nabla f\left(y\right)=\frac{1}{n}\sum_{j=1}^{n}\nabla f_{j}\left(\omega_{t}^{n}\right)$ exact gradient. Gradient updates are then corrected using *y* and ${\bar{g}}_{y}$ by Equation (2).

(2) $$g^{t}=\nabla f_{i_{t}}\left(\omega \right)-\nabla f_{i_{i}}\left(y\right)+{\bar{g}}_{y}$$

This method avoids the need for additional storage in iterative traversals algorithms, such as SAGA. Still, this method requires gradient evaluation of the entire dataset at each iteration, which is computationally expensive [38]. To solve this problem and obtain accelerated computation, the variance reduction method proposed in this paper accumulates the average gradient vector at each iteration, then uses this vector to solve the gradient for the next iteration, avoiding costly training when having to use iterative loops over the entire dataset. These accumulated average gradient vectors have no other significant overhead when the machine learning algorithm is executed. Let $\pi_{n}$ denote the random permutation of the data index $\left\{1,2,\ldots ,n\right\}$ of random permutations, $\pi_{n}^{J}$ denoting the data indexes selected with *J* length. The update rule for the variance reduction method in this paper is indicated by Equation (3).

(3) $$\omega_{n+1}^{t+1}=\omega_{n+1}^{t}-\lambda_{4}\left(\nabla f_{i_{t}}\left(\omega_{n+1}^{t}\right)-\nabla f_{i_{t}}\left({\bar{\omega }}_{n}\right)+{\bar{g}}_{n}\right)$$

where $i_{t}=\pi_{n+1}^{t},{\bar{\omega }}_{n},\;\mathrm{and}\;{\bar{g}}_{n}$ are defined as the average number of iterations, the model parameters, and the gradient values. Where the model parameters and gradient values are derived from the following Equation (4).

(4) $${\bar{\omega }}_{n}=\frac{1}{m}\sum_{i=1}^{m}\omega_{n}^{i},\;{\bar{g}}_{n}=\frac{1}{J}\sum_{j=1}^{J}\nabla f_{\pi_{n}^{t}}\left(\omega_{n}^{i}\right)$$

The variance reduction in the mean gradient vector-based variance reduction $E\left(\nabla f_{i_{t}}\left({\bar{\omega }}_{n}\right)-{\bar{g}}_{n}\right)\neq 0$ is the same as the other variance reduction methods described in the previous section, making it easy to theoretically prove the convergence of the variance reduction method in this paper.

In summary, this paper’s method does not require any additional storage other than storing and updating the average gradient vector. Each iteration requires only the historical gradient values of the previous *j* iterations of that round to participate in the calculation. The variance reduction method in this paper is shown in Algorithm 1.
**Algorithm** **1:** Mean gradient vector-based variance reduction**Parameters:** learning rate λ**initialize:**$\omega ,\bar{\omega }\;\mathrm{and}\;\bar{g}$ using plain SGD for 1 epoch 1:**while** not converged **do** 2:      initialize variables to accumulate averages over an epoch: $\tilde{\omega }=\tilde{g}=0$ 3:      **for** iteration = 1, 2,…,t **do** 4:            Sample $i_{t}\in \left\{1,\ldots ,m\right\}$ without replacement 5:            Update ω according to Equation (3): $\omega \leftarrow \omega -\lambda \left(\nabla f_{i_{t}}\left(\omega \right)-\nabla f_{i_{t}}\left(\bar{\omega }\right)+\bar{g}\right)$ 6:            Update running averages: $\tilde{\omega }\leftarrow \tilde{\omega }+\omega ,\tilde{g}\leftarrow \tilde{g}+\nabla f_{i_{t}}\left(\omega \right)$ 7:      Set $\bar{\omega }$ and $\bar{g}$ for next epoch: $\bar{\omega }=\tilde{\omega }/m,\bar{g}=\tilde{g}/m$ 8:**return**$\bar{\omega }$ and $\bar{g}$

## 4. Distributed Implementation

This paper’s distributed machine learning cluster is implemented based on distributed Tensorflow. Cluster nodes are divided into servers and working nodes, this paper uses data parallelism, but not model parallelism.

The server receives the model parameters uploaded by each worker node and aggregates them together as global processing parameters. All the updated global parameters are downlinked to the worker nodes as initial parameters for the next iteration. It is important to note that the different servers are independent of each other. The communication service occurs in the interaction between the server and the worker node, i.e., each server maintains the corresponding worker node’s model parameters. The parameters are not shared between different servers. In distributed TensorFlow, the communication protocol [31] between servers and worker nodes is based on gRPC, which is not discussed in this paper. When a worker node pushes model parameters to the server, the server aggregates these model parameters. The server then averages them, and these averages are used as the latest global parameters and pushed to all worker nodes for subsequent iterations. For example, in the synchronous approach, with a working node and each working node generating *b* parameters, the server’s global parameters are processed as $\sum_{a}\sum_{b}\omega_{t}^{ij}/ab$. To better manifest the acceleration effect of variance reduction in this paper using the asynchronous approach, the server’s global parameters are calculated using average value.

Worker nodes: The latest global parameters extracted from the server by each worker node are cached as initial parameters. These initial parameters are stored in the worker nodes instead of local parameters to become new local parameters. The worker nodes and cluster nodes are set to perform machine learning tasks asynchronously by extracting the server’s parameters via gRPC messaging. The worker node starts the machine learning model iterative computation and is trained by randomly selecting some training samples to calculate the local gradients. While performing iterative computation, all worker nodes are independent of each other. When this iteration ends, the worker nodes push the trained machine learning model parameters of this iteration to the server. Due to the variance reduction technique, in our proposed DisSAGD, in the working node, we change the gradient update of the traditional SGD as shown in Algorithm 2.
**Algorithm** **2:** Node’s work  1:**while** true **do**  2:      Send a pull request to a server  3:      **while** true **do**  4:            **if** receive a copy of the global parameters from the server **then**  5:           Cache it as the new local parameters. i.e., ω  6:           $\tilde{g}\leftarrow \tilde{g}+\nabla f_{i_{i}}\left(\omega \right)$  7:           **for** iteration = 1, 2,…,t **do**  8:                 Randomly sample a non-negative umber *i* with i∈{0,1,2,…,m}  9:                 $\omega_{n+1}^{t+1}=\omega_{n+1}^{t}-\lambda_{4}\left(\nabla f_{i_{t}}\left(\omega_{n+1}^{t}\right)-\nabla f_{i_{t}}\left({\bar{\omega }}_{n}\right)+{\bar{g}}_{n}\right)$            Send $\omega^{t}$ to a server.

**Figure 1:**
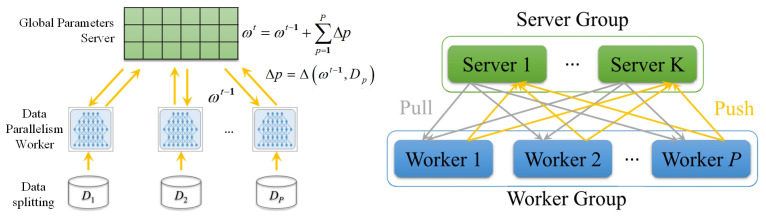
Distributed topology diagram.

The workflow of the distributed scenario implemented in this paper is described as follows: firstly, the working nodes extract the server-side parameters. Next, the basic calculation of the gradient is performed by the worker nodes. These updated model parameters are then uploaded to the corresponding server and averaged over all the newly uploaded model parameters as the latest global parameters. Finally, these new global parameters are downlinked to the corresponding work nodes. For example, the data is distributed over *P* worker nodes, then the goal of these nodes is to minimize their global objective function. We Specify that the *P* working nodes can only communicate with the corresponding servers, and the distributed topology is illustrated in Figure 1. The data index set $\left\{1,2,\ldots ,n\right\}$ is decomposed into disjoint subsets $\left\{\psi_{s}\right\}$, each $\psi_{s}$ denoting the indexes of the data stored on sth server. The objective function is shown in Equation (5).

(5) $$f\left(x\right)=\frac{1}{p}\sum_{i=1}^{p}\sum_{j\in \psi_{s}}f_{ij}\left(\omega \right)$$

The variance reduction method detailed in this paper is easy to implement for asynchronous distribution because it does not involve calculating the complete gradient of the objective function, as opposed to SVRG. Our method only needs to update the gradient’s average value on the server side at the end of each distributed iteration. We can increase the amount of communication between the server and the local nodes to achieve the fast and stable convergence of the global model.

The specific implementation of DisSAGD is given in Algorithm 3, where each iteration is performed to execute DisSAGD (the external for loop in line 2 starts). Samples are randomly sampled at each model training iteration (internal for loop starting from line 6), and parameter updates are made using a decreasing variance gradient (lines 8 and 9). DisSAGD starts the server to create the distributed cluster first, then starts the master-worker node, and coordinates the work between each remaining worker node, which includes tasks such as initialization, synchronization waiting, etc.; all worker nodes are passed model parameters via the gRPC update notification. Once a working node receives a message from the server, it extracts the server’s global parameters and starts the computation for this iteration. The random sampling strategy is performed on the worker nodes; we discuss this optimization strategy as follows: the working nodes perform model training by calculating the variance reduction gradient (line 8) and updating the local gradient (line 9) with the latest acquired global gradient. After the update, the new parameters on the local working node are uploaded to the corresponding server.
**Algorithm** **3:** DisSAGD  1:**Initialize:**${\tilde{\omega }}^{0}$//Pull the global parameters by all workers from the servers  2:**for** iteration = 1, 2,…,t **do** // Asynchronously update the parameters by all workers  3:      $\tilde{\omega }={\tilde{\omega }}^{t-1}$  4:      $\tilde{f}\left(\tilde{\omega }\right)=\frac{1}{n}\sum_{i=1}^{n}\nabla f_{i}\left(\tilde{\omega }\right)$  5:    $\omega_{0}=\tilde{\omega }$  6:      **for** iteration = 1, 2,...,t **do**  7:         Sample $i_{t}\in \left\{1,\ldots .,m\right\}$  8:         ${\tilde{g}}_{t}\leftarrow {\tilde{g}}_{t}+\nabla f_{i_{i}}\left(\omega \right)$ //Variance reduced gradient ${\bar{g}}_{t}={\tilde{g}}_{t}/m$  9:         $\omega_{t+1}\leftarrow \omega_{t}-\lambda \left(\nabla f\left(\tilde{\omega }\right)-f_{i_{t}}\left(\omega \right)+{\bar{g}}_{t}\right)$//Update the parameters with variance reduction gradient10:      ${\tilde{\omega }}^{t+1}=\omega_{t}$//Push the newly parameters to the servers

As mentioned above, due to the non-global gradient $g_{t}$ in the standard SGD, this leads to variance and inevitably slows down loss function’s convergence. In contrast, the use of variance reduction in our proposed DisSAGD allows for efficient use of historical gradients [3], which reduces the variance. The working nodes in the distributed scenario we implemented are asynchronous, so the working nodes’ update parameters are chosen randomly. The variance of the gradient on each node is effectively reduced after global iteration.

## 5. DisSAGD Optimization

### 5.1. Adaptive Learning Rate

Many methods use variance reduction to accelerate model training by setting the learning rate to a constant one. While a constant learning rate is also good for L-smoothing [39] and γ-strongly convex [33] objective functions, a learning rate that is adaptive to the training environment is more likely to accelerate the convergence rate of model training; therefore, in this paper, an acceleration factor σ is introduced to guide the learning rate variation of DisSAGD, as shown in Equation (6).

(6) $$\lambda_{t+1}=\lambda_{t}+\sigma$$

The adaptive learning rate we designed consists of two components: a constant component (constant) and an adaptive acceleration factor. Its constant component can be set based on empirical values. The addition of the adaptive acceleration factor accelerates the convergence of DisSAGD because the learning rate decay problem mainly allows the learning rate to train with a higher learning rate in the early stage, which allows the model to iterate quickly and train to converge. In contrast, in the later stage, to ensure that the model does not skip the optimal point, it is necessary to iterate the learning rate with a reduced value. The traditional solution is mainly to set the learning rate from a high to a low rate in order to train gradually. We propose an adaptive learning rate that increases the acceleration factor when the gradient is solved far from the optimal value, thus causing the gradient to nose-dive rapidly toward the saddle point. We set the upper limit of this acceleration factor to ensure model training stability. We also consider that the objective function $f_{i}$ of the model is generally convex, i.e., L-smooth, and $\nabla f_{i}$ does not change outside the iteration range. We set σ close to zero when the gradient approximates the saddle point. This setting of the acceleration factor allows DisSAGD to converge at a constant rate and without additional overhead.

**Figure 2:**
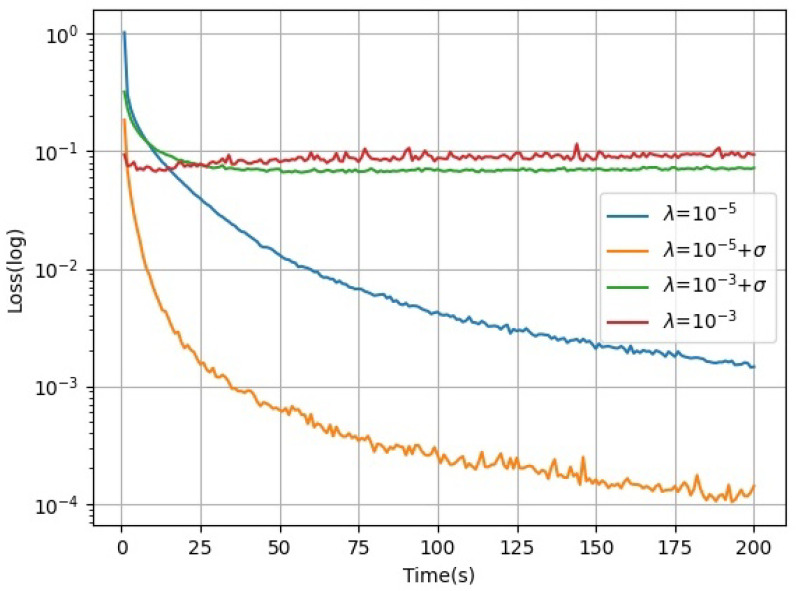
Model convergence of different learning rates.

Learning rates with acceleration factors were evaluated and tested on independent working nodes using acceleration factors for linear regression tasks. The dataset used is SVHN [40], an open-source street view door number dataset from Google, as it is a classical dataset that works with linear regression tasks. The test results are presented in Figure 2, where the horizontal axis is the training time and the vertical axis is the loss function. To illustrate the effectiveness of the acceleration factor, a fast learning rate $\lambda =10^{-3}$ is artificially set, and it is clear from Figure 2 that the acceleration factor is still effective in accelerating the algorithm even if the fixed value is too heavy. The learning rate with the acceleration factor in model training converges faster than those without the acceleration factor. The learning rate in a small fixed-value fraction has a significant convergence effect on the machine learning model. The effect of the acceleration factor on the convergence of the machine learning algorithm can even be seen in the short term.

### 5.2. Adaptive Sampling Strategy

Since the working nodes of DisSAGD need to use the sampling strategy for model training update parameters for each iteration, it is important to consider how many random samples of training data are sampled for model training. As shown in Algorithm 3, *t* denotes the number of updates per iteration. First, a very large number of *t* cannot be provided in order to avoid the working nodes spending a lot of time on model training in each global iteration. Second, *t* cannot be set to a small number either because DisSAGD must perform an additional number of global iterations to achieve global convergence. Performing iterations requires the average gradient of the loss function, and a small *t* value means that a large number of average gradients need to be computed, which leads to more time consumption. Since the runtime environments or hardware configurations of the working sections in distributed scenarios are different, many schemes asynchronously execute the working nodes. Although asynchronous communication allows for delays, when the allowed delay time is exceeded, the faster training worker nodes in the same iteration must wait for the slower worker nodes until the server updates the global parameters for all the worker nodes. Such a waiting time greatly increases the convergence time of DisSAGD. A method to reduce potential waiting time can be used to dynamically adjust the sampling strategy. We propose an adaptive dynamic sampling strategy that dynamically adjusts *t* to solve the problem. After each iteration, the new local parameters will be pushed to the server, which will check whether all the working nodes acquire the global parameters. If any working nodes finish training faster than other nodes, they need to wait for other slower working nodes. Fast working nodes scale up to *t* to t+δ, where δ is a non-negative integer; we set δ=0.035t. In this way, fast-training working nodes sample more training data samples at a time; therefore, fast-training working nodes spend more time computing parameters waiting for the slower working nodes.

**Figure 3:**
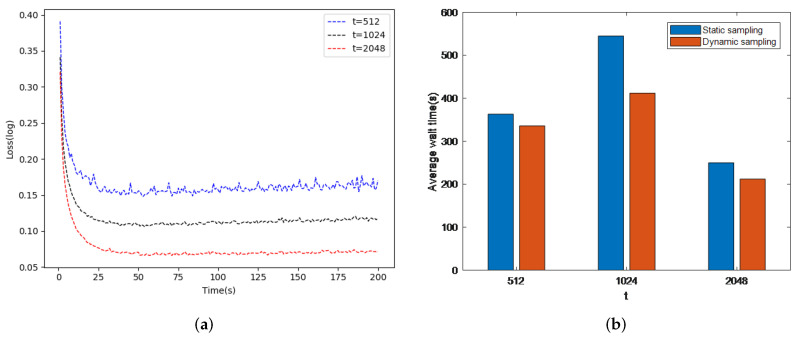
DisSAGD convergence and waiting time during iteration ((**a**) is DisSAGD convergence and (**b**) is waiting time during iteration).

As a result, the waiting time between the working nodes is reduced. In this paper, we evaluated the working nodes by changing the value of *t*. The dataset was SVHN and we used our proposed adaptive learning rate. As shown in Figure 3a, it is clear that the adjusted *t* results in a quick objective function convergence; therefore, the adjusted *t* reduces the loss function. Another evaluation test was performed to compare the waiting time for a cluster of six nodes. Figure 3b shows that the average waiting time between nodes can be reduced by varying *t*; we set the delay to 0.035 s (Ref. [41]). It is clear that our proposed strategy reduces the average waiting time during the global iteration and this time trade-off increases the accuracy of model training.

## 6. Performance

In this section, we evaluate the performance of DisSAGD using the anomaly detection and the classification on two datasets. We consider the anomaly detection with a loss function modeled as MSE, as shown in Equation (7).

(7) $$\mathrm{mse}=\frac{1}{N}\sum_{i=1}^{N}{\left(x_{i}-y_{i}\right)}^{2}$$

where *x* is the training data, *y* is the machine model output value, and *N* is the size of the training data. The KDDcup99 dataset [42] is the classical dataset for the anomaly detection problem and contains 463,715 samples with 41 dimensions each.

We consider the classification problem with a loss function model, as shown in Equation (8).

(8) $$\min-\frac{1}{N}\sum_{i=1}^{N}\left(y_{i}\log\frac{1}{1+e^{-\omega x_{i}}}+\left(1-y_{i}\right)\log\left(1-\frac{1}{1+e^{-\omega x_{i}}}\right)\right)$$

The CIFAR-100 is a representative dataset for the machine learning classification problem [43], consisting of 60,000 three-channel color images with a size of 32 * 32, divided into 20 major categories, each of which contains five subcategories, and a total of 100 subcategories. Each subclass contains 600 images, 500 of which are used for training and 100 for testing.

In this paper, the distributed cluster training platform was evaluated and tested on the Starring Hyperconverged Big Data All-in-One cluster. This cluster has 32 compute nodes, and is equipped with 1536-core CPUs. In this evaluation, *t* is set to 1024, and the distributed implementation uses distributed TensorFlow to evaluate the performance. All compared algorithms are based on the distributed TensorFlow implementation.

In this paper, the following algorithms were used to compare the training performance of machine learning models.

PetuumSGD: a distributed SGD implemented using an asynchronous approach, the learning rate of PetuumSGD decays by a fixed factor of 0.95 at the end of each iteration.

SSGD: a recently proposed distributed SGD, which uses variance reduction techniques [25] and asynchronous parameter update.

DisSVRG [44]: improves the random variance reduction algorithm by extending it from the original serial stand-alone version to a distributed training algorithm for large-scale datasets.

ASD-SVRG [45]: a newer distributed optimization algorithm that uses SVRG for adaptive sampling and estimates the Lipschitz constant based on the local part of the historical gradient.

DisSAGD-tricks: this paper uses all tricks of DisSAGD, such as the adaptive learning rate and sampling strategy.

DisSAGD: DisSAGD without the use of additional features.

### 6.1. Convergence Performance

**Figure 4:**
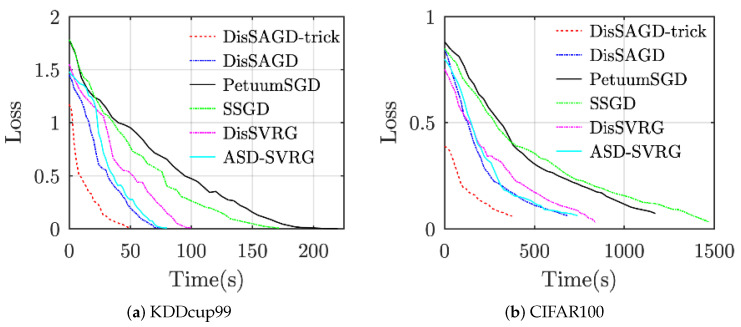
Compare convergence performance by using 4 computational nodes.

The convergence performance of the algorithm has been evaluated as shown in Figure 4. The machine learning algorithm uses asynchronous communication when the delay is set to 0.5 s. All datasets are allocated to four working nodes. As can be clearly seen from Figure 4, the method proposed in this paper outperforms the other baselines for all datasets, even though DisSAGD does not use our proposed accelerated optimization technique, which also outperforms the baseline, especially for KDDcup99. DisSAGD outperforms existing algorithms an asynchronous approach and update the parameters based on the mean gradient vector variance reduction. DisSAGD was set to use the optimization technique and only one-quarter and one-third of the KDDcup99 [42] and CIFAR-100 datasets, respectively. We found that the DisSAGD algorithm performance depends on the specific dataset, i.e., it does not exceed SSGD on KDDcup99, whereas it outperforms SSGD on Cifar100, but the overall convergence performance is better than the performance of ASD-SVRG, which significantly better than that of PetuumSGD and SSGD, and is close to that of DisSAGD (both have equal loss function values at a given time, but the performance of ASD-SVRG improves as time increases); on the other hand, the performance of ASD-SVRG is comparable to that of DisSAGD equivalent, but significantly inferior to the optimized DisSAGD (i.e., DisSAGD-tricks). The experimental results show that DisSAGD uses an asynchronous approach as well as a mean gradient vector variance reduction-based parameter update, and the learning rate with an acceleration factor acceleration the convergence, whereas the adaptive sampling strategy significantly reduces the waiting time for the server to distribute the global parameters.

### 6.2. Acceleration Effect

**Figure 5:**
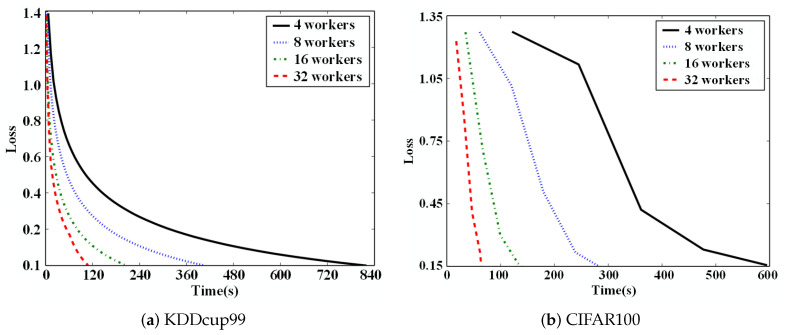
Acceleration effect with different working nodes.

To show that DisSAGD can achieve better convergence performance with different working node settings, we deployed DisSAGD under a distributed scenario with different numbers of working nodes. As shown in Figure 5, the increase in training speed of DisSAGD is approximately linear with a larger number of working nodes, and DisSAGD was 19 times faster on the KDDcup99 dataset with four working nodes when 32 working nodes are used. DisSAGD even maintains a linear speed increase when 32 workers are utilized for CIFAR-100. These converging linear speed increases are mainly attributed to the use of asynchronous methods and variance reduction. The asynchronous approach allows the working nodes to work with each other without delay, thus reducing the waiting time between working nodes. The variance reduction reduces the SGD variance at each node allowing for shorter solution times and the stable convergence of DisSAGD.

### 6.3. Waiting Time

**Figure 6:**
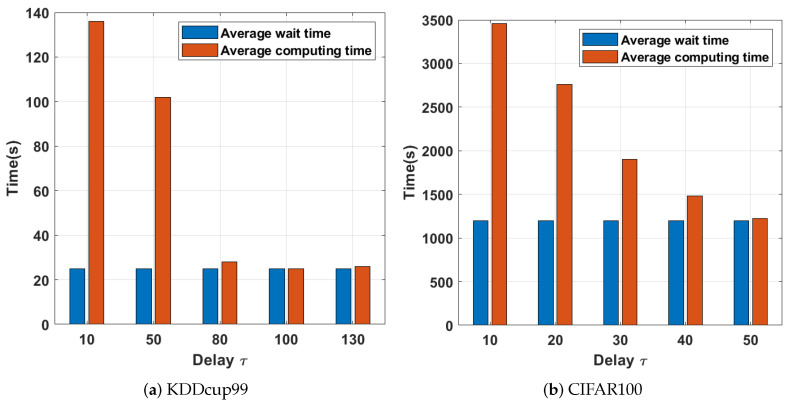
Delay time consumption with different working nodes.

As shown in Figure 6, the average waiting time between nodes with a small τ is significant. The smaller τ implies a tight connection between the working nodes, which usually causes the working nodes with fast computation to spend time idle due to the updated method. When the latency is set to 0, the server downlinks global parameters to all working nodes in each global iteration, and each node participates in the computation, thus increasing the average waiting time of nodes. When the latency increases, the waiting time decreases sharply; therefore, setting a reasonable communication delay can effectively reduce the average waiting time. Although fast working nodes have more free time to iterate, slow working nodes cannot obtain new global parameters from the server within the large latency; therefore, slow worker nodes cannot benefit from fast worker nodes. The latency, if set, needs to be set according to different situations. The coordination between the working node waiting time and the computation time needs to be considered simultaneously. Empirically, the optimal setting of the delay should minimize the total time consumption of the distributed training. For the evaluation tests in this paper, we set τ to 200 and 50 for KDDcup99 and CIFAR100, respectively, which we obtained empirically.

### 6.4. Time Difference Balance

**Figure 7:**
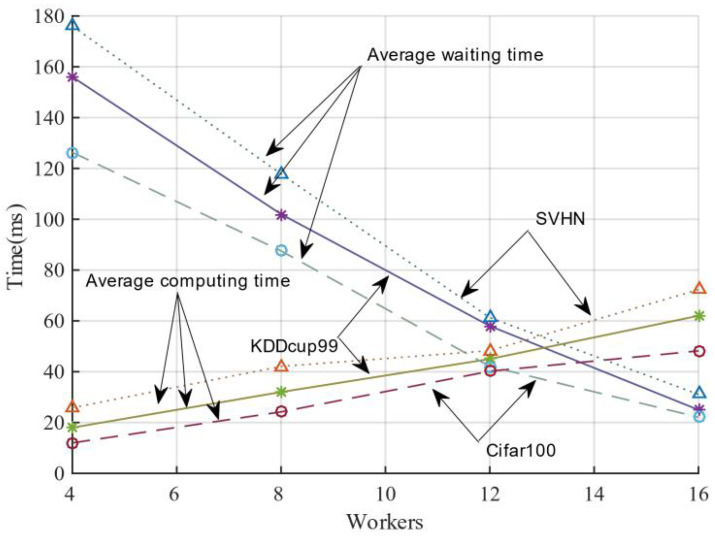
Balance between average waiting time and average computing time.

In order to investigate how many worker nodes set to interact with the server can better balance the difference between computation and waiting time, in this study, we initiated the computation of global parameters by collecting the model parameters of a specific number of worker nodes from the server and then iterated them across the distributed cluster. The results are shown in Figure 7. When the server aggregates the number of working nodes from 2 to 16, its average waiting time gradually decreases. Its average computation time gradually builds up when the number of nodes increases. Their average waiting time and average computation time intersect at a definite number of worker nodes for three datasets. In general, the average waiting time decrease is greater than the average computation time, indicating that the gain by specifying the number of working nodes is not as significant as that obtained by setting the delay time. The working nodes can be adaptively sampled for model training within the delay time, resulting in better efficiency and convergence performance.

## 7. Conclusions

In this paper, we propose a stochastic gradient descent variance reduction based on historical value for distributed implementations, which we call DisSAGD, without any complete gradient computation or additional storage. Using the nature of the loss function, we also set an adaptive learning rate to optimize DisSAGD. Further, to reduce the waiting times between working nodes, we propose an adaptive sampling strategy that allows the slower working nodes’ computational speed to catch up with the faster working nodes. Our experiments show that DisSAGD converges faster than other methods, and an improved training time can be obtained in distributed clusters.

## Figures and Tables

**Table 1:** Comparison of different working performance.

Reference	Synchronous	Fast Convergence (Training Time (s))	Stable	Small Estimation Error	Maintains Model Accuracy	Amount of Communication Data (bit)	Publication Year
Guo Y. et al. [23]	✓	329	✓	0.023	✓	518	2021
Zhou Q. et al. [24]	✕	263	✓	0.032	✓	387	2020
Amiri M. et al. [26]	✓	5768	✓	0.124	✕	29	2020
Xing E P. et al. [27]	✕	5524	✕	0.018	✓	457	2015
Zhang R. et al. [28]	✓	158	✕	0.009	✕	53	2016
Johnson R. et al. [30]	✓	144	✓	0.015	✓	36	2019
Chen A A. et al. [35]	✓	6253	✕	0.021	✕	526	2018
our paper	✓	128	✓	0.006	✓	22	

## Data Availability

Publicly available datasets were analyzed in this study. This data can be found here: http://kdd.ics.uci.edu/databases/kddcup99/kddcup99.html; http://www.cs.toronto.edu/~kriz/cifar.html; accessed on 16 July 2021.
